# Prolymphocytic Progression in Chronic Lymphocytic Leukemia Without Richter Transformation: An Eighteen‐Year Clinical‐Diagnostic Correlation

**DOI:** 10.1155/crh/9707876

**Published:** 2026-08-21

**Authors:** Harini Venkatraman Ravisankar, Kavita Umrau, Liang Cheng, Magdalena Czader, Rohit Gulati

**Affiliations:** ^1^ Department of Pathology and Laboratory Medicine, Indiana University School of Medicine, 350 W 11th Street, Indianapolis Indiana, 46202, USA, indiana.edu; ^2^ Department of Pathology and Laboratory Medicine, Brown University, Providence 02912, Rhode Island, USA, brown.edu; ^3^ Department of Pathology and Laboratory Medicine, Indiana University School of Medicine, 350 W 11th Stree Room 5048, Indianapolis Indiana, 46202, USA, indiana.edu

**Keywords:** accelerated CLL, B-prolymphocytic leukemia, chronic lymphocytic leukemia, diffuse large B-cell lymphoma, genetics of CLL, prolymphocyte, prolymphocytes in CLL, prominent proliferation centers, Richter transformation, small lymphocytic lymphoma

## Abstract

Chronic lymphocytic leukemia (CLL) is an indolent neoplasm of small, mature B lymphocytes characterized by clumped chromatin and scant cytoplasm. A minor subset of neoplastic cells may exhibit prolymphocytic morphology, with prominent nucleoli and moderate cytoplasm. An increased proportion of prolymphocytes in peripheral blood often correlates with an accelerated or aggressive disease course, distinct from Richter transformation (RT), which more commonly manifests as diffuse large B‐cell lymphoma and requires intensified therapy. We report an 18‐year clinical course of CLL in a 57‐year‐old man that evolved to prolymphocytic progression without transformation to large‐cell lymphoma. Serial morphological assessments revealed a steady increase in prolymphocytes compatible with prolymphocytic progression in CLL and excluded RT. At the final assessment, 70%–80% of circulating lymphocytes exhibited prolymphocytic morphology, fulfilling criteria for prolymphocytic progression and reclassification as accelerated CLL. Accompanied immunophenotypic and IgH gene rearrangement findings confirmed CLL clonality. This case underscores the diagnostic and prognostic importance of recognizing prolymphocytic progression and integrating morphology with immunophenotypic and molecular data in the longitudinal management of CLL.

## 1. Introduction

Chronic lymphocytic leukemia (CLL) is an indolent mature B‐cell lymphoproliferation with a higher incidence with advancing age. The diagnosis of CLL relies on integration of lymphocyte morphology, immunophenotypic profile, and evidence of clonality. A monomorphic population of small lymphocytes with mature clumped “soccer ball‐like” chromatin and scant cytoplasm is typical. Immunophenotypically, the expression of B‐cell markers, especially with dim CD20 and dim surface light chains, along with expression of CD5 and CD23, is characteristic [1, 2].

Some CLL may deviate from classic morphology or immunophenotype. Cells may show a moderate amount of cytoplasm or lack typical clumped chromatin. Further, flow cytometry may reveal bright light chains or CD5/CD23 expression. A combination of slightly atypical morphology with characteristic immunophenotype or vice versa is still compatible with CLL (considered with atypical features), provided other close differential diagnoses, such as mantle cell lymphoma (MCL), are excluded [2].

Prolymphocytes are intermediate in size with moderate cytoplasm, round nuclei with mature chromatin, and prominent central nucleolus. Prolymphocytes are common in B‐lymphoproliferative disorders such as B‐prolymphocytic leukemia (B‐PLL) and hairy cell leukemia‐variant (HCL‐v) (now grouped under “splenic B‐cell lymphoma/leukemia with prominent nucleoli”) [1]. In CLL, a small proportion of cells (< 15%) may show prolymphocytic morphology in peripheral blood and bone marrow. Bona fide cases of CLL with higher (usually > 55%) prolymphocytes are not very common and are best described as a prolymphocytic progression. Prolymphocytic progression in CLL is biologically distinct and managed differently from Richter transformation (RT) and splenic B‐cell lymphoma/leukemia with prominent nucleoli. Increased prolymphocytes in peripheral blood in CLL correlate with an aggressive disease course and in lymph nodes with the “histologically aggressive” CLL manifesting with enlarged proliferation centers [1, 3, 4].

Herein, we present a case of CLL followed for nearly 2 decades with prolymphocytic progression in peripheral blood paralleling progressive clinical deterioration and discuss the diagnostic nuances and clinical implications of this phenomenon.

## 2. Case Presentation

**FIGURE 1:**
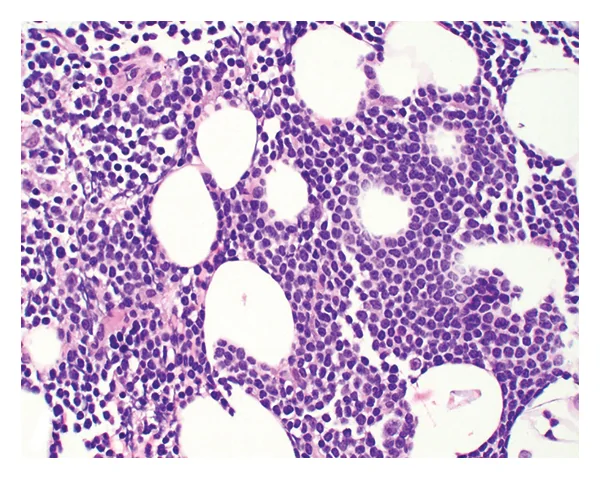
Left axillary lymph node biopsy 12 years after initial diagnosis of chronic lymphocytic leukemia (CLL) (Hematoxylin and eosin (HE) stain, 50X) shows sheets of mainly small lymphocytes and occasional intermediate‐sized lymphocytes with prominent nucleoli (prolymphocytic cells).

A 57‐year‐old male with a past medical history of diabetes mellitus and coronary artery disease has incidental lymphocytosis on a routine hemogram. The complete blood counts (CBC) revealed total white blood cells (WBC) of 15.8 × 10^9^/L (normal range: 1.5–8.0 × 10^9^/L) with absolute lymphocyte count (ALC) of 9.9 × 10^9^/L (normal range: 1.0–3.3 × 10^9^/L), hemoglobin (Hgb) 15.8 × 10 g/L (normal range: 11–16 × 10 g/L), and platelet count (PLT) 182 × 10^9^/L (normal range: 150–450 × 10^9^/L). Peripheral blood smear evaluation revealed small mature lymphocytes and numerous smudge cells. A physical exam revealed bilateral cervical and axillary lymphadenopathy, and subsequent CT imaging showed mild abdominal and pelvic lymphadenopathy (largest mesenteric lymph node measured 1.6 × 2.8 cm, largest right iliac chain lymph node measured 2.3 × 2.7 cm, and largest left iliac chain lymph node measured 2.8 × 3.1 cm) and no splenomegaly. The patient was on observation with no treatment. Three years later, the ALC increased to 99.6 × 10^9^/L, the Hgb slightly decreased to 12.8 × 10 g/L, and PLT remained within normal limits. CT imaging revealed diffuse lymphadenopathy involving bilateral axillary, retroperitoneal, mesenteric, pelvic, and inguinal regions (conglomerate retroperitoneal nodal disease measured 7.6 × 11.2 cm, the conglomerate right pelvic nodal mass measured 9.1 × 17.2 cm) and mild splenomegaly with a subcentimeter hypoattenuating ill‐defined lesions. The patient was treated with five cycles of fludarabine, achieved complete remission, and then four cycles of rituximab maintenance. After stabilization from chemotherapy, CBC showed WBC 5.3 × 10^9^/L, ALC 1.06 × 10^9^/L, Hgb 10.6 × 10 g/L, and PLT 140 × 10^9^/L. The patient was stable, on observation, for the next 6 years and subsequently developed lower extremity swelling. The imaging showed an interval increase in the size of the lower retroperitoneal lymph nodes encasing the aorta. CBC showed no lymphocytosis. Due to the excellent performance status and radiographic‐only disease, the patient was followed without chemotherapy over the next 3 years with an interval increase in the size of lower retroperitoneal lymph nodes. The spleen size and its hypoattenuating lesions remained stable. Subsequently, the patient showed hematological progression with mild lymphocytosis (WBC 19.5 × 10^9^/L, ALC 14.2 × 10^9^/L) and mild thrombocytopenia. The patient underwent 3 cycles of bendamustine and rituximab, while ALC declined to 2.2 × 10^9^/L. Radiology revealed progressive retroperitoneal lymphadenopathy and stable splenomegaly. At this point, the peripheral blood smear showed predominantly small mature lymphocytes and occasional prolymphocytes. Needle core biopsy of the left axillary lymph node showed sheets of predominantly small lymphocytes and occasional intermediate‐sized cells with distinct nucleoli (Figure 1).

The axillary node was selected for biopsy in preference to the dominant retroperitoneal/periaortic mass owing to the critical anatomical relationship of the latter with major vascular structures and the patient’s elevated procedural bleeding risk while on concurrent dabigatran anticoagulation for atrial fibrillation. Although the retroperitoneal node represented the dominant site of radiological progression and would ordinarily have been the preferred sampling target, PET/CT‐guided biopsy was not performed. The axillary biopsy demonstrated a proliferative index of approximately 40% by Ki‐67 immunohistochemistry, fulfilling criteria for histologically aggressive CLL/SLL as subsequently defined in the fifth edition of the WHO Classification of Hematolymphoid Tumors (WHO), though this diagnostic framework was not yet in use at the time the biopsy was rendered.

**FIGURE 2:**
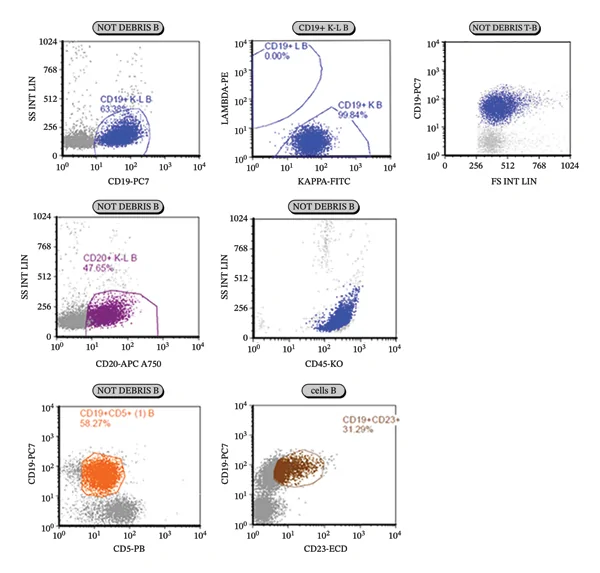
Flow cytometry on specimen collected 12 years after initial diagnosis (morphology in fig. A) reveals a clonal B‐cell population with expression of CD19+, CD20 (dim+), CD5+, and CD23 (partial+), with dim surface kappa light chains characteristic of CLL.

Flow cytometry revealed a monoclonal B‐cell population expressing CD19, dim CD20, CD5, and partial CD23 positivity, with dim surface kappa light chain consistent with a diagnosis of CLL (Figure 2). Also, the B‐cell population was negative for other markers such as CD34, CD33, CD10, CD3, CD4, CD7, and CD8 and lambda. Flow cytometric analysis was performed (for antibodies CD34, CD33, CD19, CD20, CD5, CD22, CD23, CD10, HLA‐Dr, CD71, Kappa, Lambda, CD3, CD4, CD5, CD7, and CD8 antibodies) using clinically validated Navios and Gallios instruments (Beckman Coulter, CA, USA). Data were subsequently analyzed using FCS Express software (De Novo Software, CA, USA).

**FIGURE 3:**
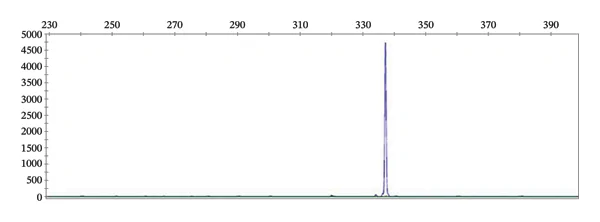
The IgH gene rearrangement analyses by PCR on specimen in Figure 1 shows amplification peak located at 337.09 bp.

PCR‐based IgH gene rearrangement analyses showed a monoclonal product of 337.09 bp (Figure 3). The DNA was extracted using QIAGEN DNA extraction kit and per manufacturers protocols. The DNA was amplified using InVivoScribe (San Diego, CA) primer kits. The PCR product was analyzed by capillary electrophoresis using Applied Biosystems 3500 xL genetic analyzer. HLA‐DQa gene was PCR amplified as an internal control. Appropriate positive and negative controls were also included in each assay. Target (product range in bp): FR1‐JH (310‐360 bp); FR2‐JH (250‐295 bp); FR3‐JH (100–170). Amplification controls: 84, 96, 200, 300, 400, and 600 bp.

**FIGURE 4:**
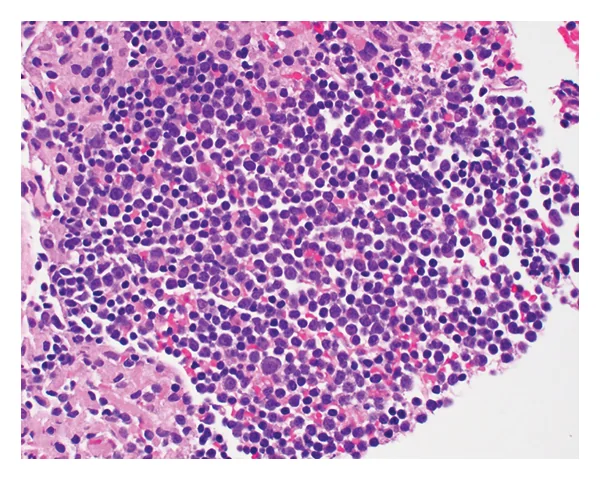
Left retroperitoneal lymph node biopsy (HE, 50X) performed 16 years following initial diagnosis shows a mix of small mature lymphocytes and frequent prolymphocytes.

**FIGURE 5:**
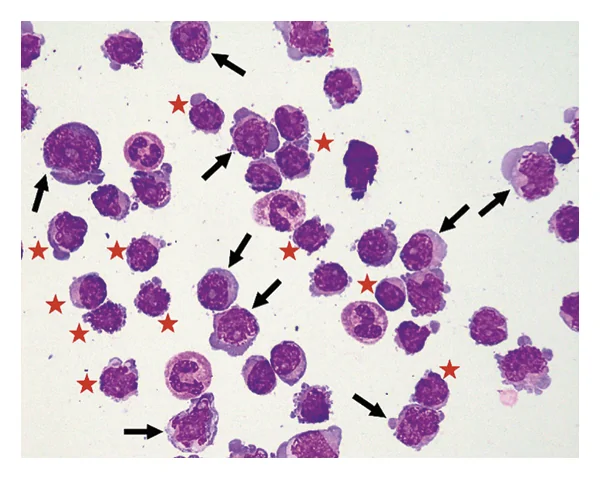
Left retroperitoneal lymph node aspirate cytospin (Wright Giemsa stain, 100X) performed 16 years following initial diagnosis shows a mix of small mature lymphocytes (annotated with “star” symbol in Figure 5) and frequent prolymphocytes (annotated with “arrow” symbol in Figure 5).

Following the lack of response to bendamustine, salvage fludarabine and rituximab resulted in an interval decrease in retroperitoneal lymphadenopathy (largest retroperitoneal nodal mass measures 4.7 × 7.4 cm, previously measured 6.2 × 10 cm). One year later, the patient experienced hematological (WBC 27.5 × 10^9^/L, ALC 21.7 × 10^9^/L, Hgb 9.7 × 10 g/L, and PLT 56 × 10^9^/L) and radiological relapse. Ibrutinib was initiated at a reduced dose (140 mg daily) due to bleeding‐related adverse effects, including petechiae, hemoptysis, and melena. Dose attenuation also accounted for concurrent dabigatran therapy for atrial fibrillation, which was subsequently discontinued after the first episode of melena to mitigate the additive bleeding risk from the known ibrutinib–dabigatran interaction. Despite the dose reduction, the patient exhibited a partial radiologic response characterized by decreased lymphadenopathy (right iliac lymph node reduced to 3.5 cm) and complete resolution of splenomegaly. Three years later, the patient relapsed with progressive lymphocytosis (ALC: 161 × 10^9^/L), thoracic and abdominal lymphadenopathy, and massive splenomegaly (25 cm). A needle core biopsy of the left retroperitoneal lymph node showed a mix of small mature lymphocytes with frequent intermediate‐sized prolymphocytic cells with prominent nucleoli and rare larger cells (Figures 4 and 5).

**FIGURE 6:**
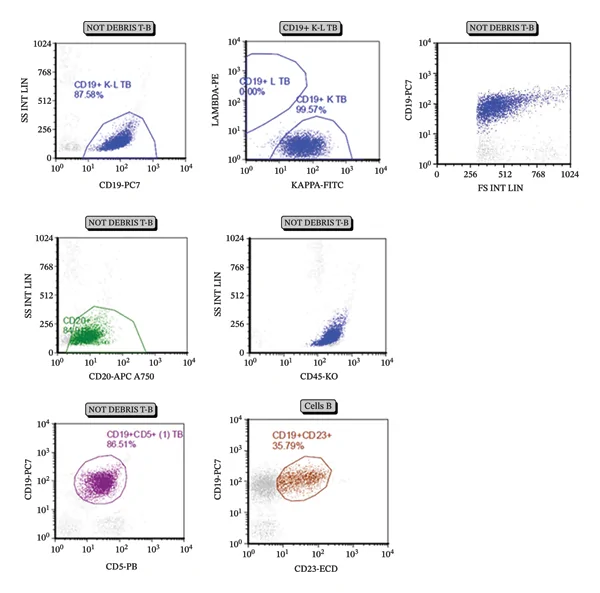
Flow cytometry on specimen collected 16 years after diagnosis shows immunophenotypic features compatible with CLL, as noted previous flow in Figure 2, with subtle increase in kappa light chain intensity.

There was no evidence of large‐cell transformation. Immunohistochemical stains for cyclin D1, c‐MYC, and p53 were negative. The absence of cyclin D1 expression by immunohistochemistry provided additional evidence against the leukemic variant of MCL. Although FISH and conventional cytogenetics were not performed, a recognized limitation of this case, negativity for cyclin D1, in conjunction with the characteristic CLL immunophenotype effectively excluded MCL. Flow cytometry showed similar findings to those previously reported with a notable increase in the intensity of kappa light chains (Figure 6).

**FIGURE 7:**
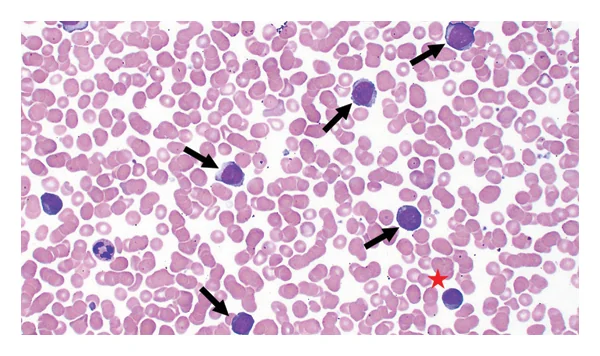
Peripheral blood smear (Wright Giemsa, 100X) 18 years following initial diagnosis of CLL showed frequent larger cells with prolymphocytic morphology (annotated with arrows) and rare small lymphocytes (annotated with “star” symbol).

This evaluation confirmed the diagnosis of CLL and excluded RT. The patient received venetoclax 400 mg orally with resolution of lymphocytosis (WBC 3.8 × 10^9^/L, ALC 2.6 × 10^9^/L, Hgb 8.7 × 10 g/L, and PLT 47 × 10^9^/L) and radiological response with complete resolution of splenomegaly. After a few months, the patient achieved complete remission. However, venetoclax was discontinued due to clinical symptoms including left upper quadrant abdominal discomfort, significant fatigue, and recurrent episodes of bruising. The patient relapsed after four months off‐therapy with diffuse lymphadenopathy, splenomegaly, and prominent lymphocytosis (ALC 46.6 × 10^9^/L). Peripheral blood smear showed frequent prolymphocytes (70%–80%) with prominent nucleoli and rare small lymphocytes (Figure 7) meeting criteria for prolymphocytic progression by both WHO (> 15%) and The International Consensus Classification of Myeloid and Lymphoid Neoplasms (ICC) (> 55%) thresholds [1, 5].

**FIGURE 8:**
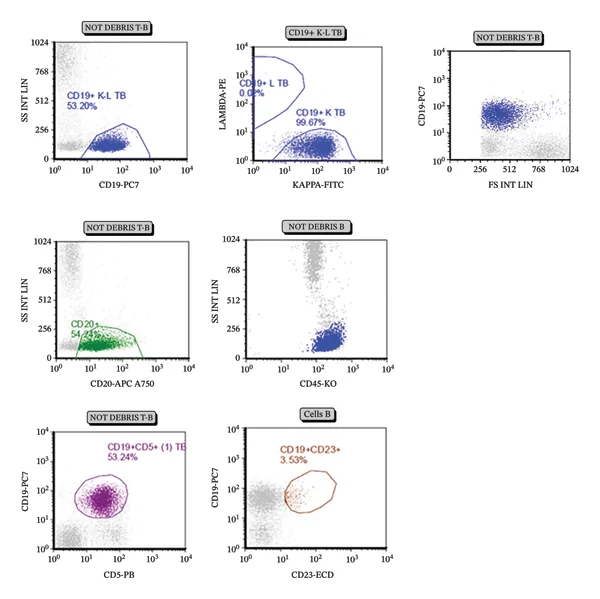
Flow cytometry on peripheral blood 18 years after initial diagnosis showed characteristic CLL immunophenotype, with moderately increased kappa light chain intensity and decreased CD23 intensity compared to prior studies (Figure 2 and 6).

Flow cytometry of peripheral blood showed a clonal B‐cell population expressing CD19, dim CD20+, CD5+, and CD23 (minimal+) but with moderate (increased) intensity of kappa light chains (Figure 8).

**FIGURE 9:**
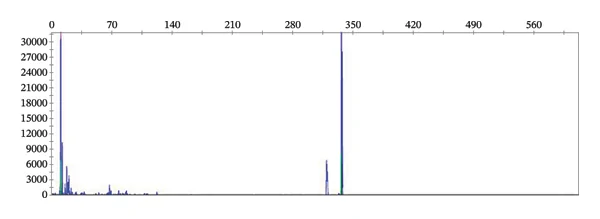
The IgH gene rearrangement analyses by PCR on specimens Figure 7 show an amplification peak located at 335.9 bp (essentially the same as noted previously in Figure 3).

An IgH gene rearrangement analysis showed a peak located at 335.9 bp, essentially the same as noted previously (Figure 9).

**FIGURE 10:**
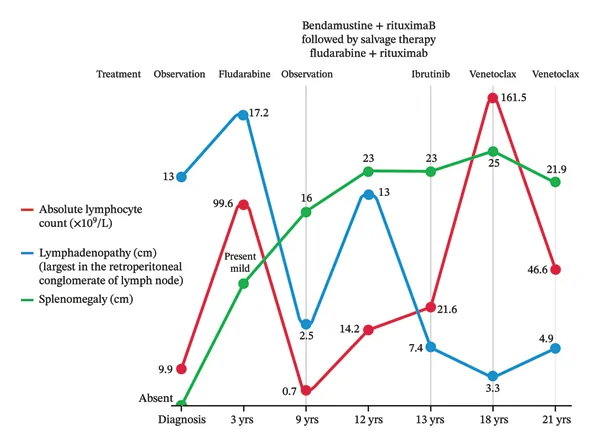
Line graph representation of absolute lymphocyte count, clinical presentation (lymphadenopathy and splenomegaly), and therapeutic intervention from the time of initial diagnosis of CLL.

Patient’s complete clinical course is summarized as a line graph in Figure 10.

No further chemotherapeutic intervention was attempted, and the patient died 1 month later.

## 3. Discussion

Increased prolymphocytic population of B‐lineage in peripheral blood warrants distinctions between B‐PLL, HCL‐v (both now grouped under splenic B‐cell lymphoma/leukemia with prolymphocytic morphology in current WHO), and CLL with prolymphocytic progression (accelerated CLL). Further in CLL, tissue evaluation of a dominant lesion to exclude potential RT (to diffuse large B‐cell lymphoma, classic Hodgkin lymphoma, or rare forms like Plasmablastic lymphoma) is essential, as the distinction carries direct therapeutic consequences. Detailed immunophenotyping and genetics studies, including karyotyping, FISH, *IGHV* gene hypermutation, methylation status, and molecular alterations, specifically *MYC, TP53, NOTCH1*, and *SF3B1*, have added significantly to our understanding of CLL prognosis and progression potential. It bears emphasis, however, that the absence of comprehensive molecular data as in the current case, where FISH, *IGHV* mutation status, and next‐generation sequencing were not obtained, does not preclude a confident morphological and immunophenotypic diagnosis, provided that a characteristic CLL immunophenotype and clonal stability are rigorously documented across serial assessments. Morphological evaluation of peripheral blood and lymph node tissue remains the cornerstone of diagnosis [3, 6–8].

The revised Fourth edition of WHO listed B‐PLL as a distinct entity supported by detailed gene expression profiling data. B‐PLL presents de novo with > 55% prolymphocytes in peripheral blood, splenomegaly, and minimal lymphadenopathy and lacks a specific defining immunophenotype. Key exclusions include the leukemic variant of MCL (excluded by absence of cyclin D1/SOX11 and t(11; 14)), typical CLL (distinguished by the characteristically dim CD20 and light chain intensity in CLL versus B‐PLL), and HCL‐v (excluded by absence of CD103, CD11c, CD25, and CD123). While the WHO fifth edition groups B‐PLL and HCL‐v under the placeholder entity *splenic B-cell lymphoma/leukemia with prominent nucleoli*, the ICC retains B‐cell prolymphocytic leukemia as a separate entity [1, 9].

The current case is best classified as accelerated CLL, a designation that encompasses two morphologically defined subtypes in the WHO fifth edition: prolymphocytic progression, defined by > 15% prolymphocytes in peripheral blood (WHO) or > 55%, and histologically aggressive CLL/SLL, defined by large or confluent proliferation centers occupying the full 20 × field of view, or a Ki‐67 proliferative index exceeding 40% within proliferation centers on tissue sections. These two subtypes likely represent different topographical manifestations of the same underlying biological process in peripheral blood and lymph node/tissue, respectively. In our patient, the axillary lymph node biopsy at Year 12 demonstrated a Ki‐67 index of approximately 40%, fulfilling tissue‐based criteria for histologically aggressive CLL. The final peripheral blood smear at Year 18 showed 70%–80% prolymphocytes, satisfying criteria for prolymphocytic progression under both classification systems. Taken together, the longitudinal findings across both specimen types support a unified diagnosis of accelerated CLL [1, 5, 9].

Both the WHO fifth edition and the ICC acknowledge that B‐PLL, HCL‐v, and splenic red pulp B‐cell lymphoma share overlapping clinical and morphological features in the absence of defining molecular markers, and both systems anticipate further reclassification as genomic data mature. The two classifications diverge, however, in their approach to this diagnostic uncertainty. The WHO fifth edition consolidates B‐PLL and HCL‐v into a single placeholder entity—*splenic B-cell lymphoma/leukemia with prominent nucleoli*—reflecting the absence of reliable distinguishing features and acknowledging the provisional nature of the grouping. The ICC, in contrast, retains B‐cell prolymphocytic leukemia as a distinct entity, while classifying splenic red pulp B‐cell lymphoma and HCL‐v as provisional entities under “splenic B‐cell lymphoma, not otherwise specified.” In practical terms, a de novo presentation of B‐cell lymphocytosis with predominantly prolymphocytic morphology should prompt consideration of splenic B‐cell lymphoma/leukemia with prominent nucleoli per WHO criteria or B‐PLL or HCL‐v per ICC criteria. In the current case, the 18‐year CLL history with stable IgH clonality effectively excludes a de novo B‐cell neoplasm under either framework; the prolymphocytic morphology is attributable to clonal evolution within the established CLL clone rather than emergence of a new disease process [1, 9].

Increasing prolymphocytes are a harbinger of poor prognosis in CLL. More than 10% prolymphocytes is associated with *NOTCH1* mutation, CD38 expression, absent 13q deletion, and *IGHV* somatic hypermutation, which portend poor prognoses by multivariate analysis. Emerging data link NOTCH1 mutations with both prolymphocytic progression and RT, mediated through the AKT–mTOR pathway. Therefore, even modest increases in prolymphocytes (> 10%) merit attention, as they correlate with adverse genetic profiles and reduced survival. In the case of prolymphocytic progression, microscopic evaluation of suspicious areas of tissue involvement is essential to differentiate a potential RT or histologically aggressive CLL/SLL. A diagnosis of RT requires aggregates of large B‐lymphoma cells, and high proliferative activity alone should not be considered diagnostic [4, 6, 9–18].

Rare cases of composite CLL and B‐PLL have been described. While the two populations may be synchronous or metachronous, documentation of two distinct B‐cell clones is essential for the diagnosis of a composite lymphoma. Of note, multiple clones may also be seen due to clonal evolution in large‐cell transformation. Clonality may be assessed by multiple methods. *IGH* (or *IGκ/IGλ*) rearrangement by PCR reports clonality based on the size of the gene product. Multicolor flow cytometry may also identify multiple distinct B‐cell populations defined by differences in side‐scatter, intensities of various markers, and/or intensity or type of light chains. Multiple clones may be seen in composite lymphomas, large‐cell transformation, or other examples of clonal evolution. Biclonality is concerning but not diagnostic of composite lymphoma/transformation without further confirmation [1, 11, 19].

CLL frequently shows abnormalities in chromosome 13q (55% of cases), 17p (7%), and 11q (6%–18%) and trisomy 12 (12%–16%). Comparatively, B‐PLL is frequently associated with a complex karyotype (≥ 3 unrelated abnormalities, 73% of cases) and MYC alterations, including *MYC* translocations in 76% of cases‐findings more specific to B‐PLL and not characteristic of CLL. Of interest, *MYC* translocations are associated with a higher number of prolymphocytes and less frequent complex cytogenetics and del17p indicative of a distinct pathway in B‐PLL. In B‐PLL, *IGHV* genes are commonly hypermutated (79%) with frequent usage of IGHV3 or IGHV4 subgroups (89%) similar to CLL but different from other mature B‐cell lymphomas like MCL and MZL. Of interest, hypermutated IGHV is associated with a good prognosis in CLL while IGHV hypermutations do not have any prognostic impact in B‐PLL.

The genetic landscapes of CLL and B‐PLL show significant overlap—including recurrent mutations in *TP53*, *SF3B1*, *MYD88*, and *NOTCH1* which partially limits the utility of molecular analyses alone for diagnostically distinguishing between the two entities. The most diagnostically useful genetic discriminator remains *MYC* gene rearrangement, which is characteristic of B‐PLL but not CLL [6, 10].

Our patient was initially managed with chemoimmunotherapy with fludarabine‐ and bendamustine‐based regimens which were commonly used as first‐ and second‐line treatments at the time. However, treatment paradigms have since evolved to favor earlier use of targeted therapies, such as Bruton tyrosine kinase (BTK) inhibitors and BCL2 inhibitors like venetoclax. The later incorporation of ibrutinib and venetoclax in our patient’s regimen reflects this shift and is consistent with current, evidence‐based approaches to CLL management [20].

The potential contribution of prior chemoimmunotherapy to clonal evolution in this patient merits consideration. Fludarabine‐based regimens have been associated with therapy‐driven selection of high‐risk clonal subpopulations including those harboring *TP53* mutations and complex karyotypes, through preferential outgrowth of pre‐existing resistant clones rather than de novo mutagenesis [21, 22]. While a direct causal link between prior cytotoxic therapy and prolymphocytic progression cannot be established in the absence of serial cytogenetic and molecular data, it is plausible that successive lines of chemoimmunotherapy contributed to the selective expansion of the prolymphocytic clone observed at later time points. This hypothesis is consistent with the broader literature on therapy‐related clonal evolution in CLL and underscores the importance of early transition to targeted therapies, such as BTK inhibitors and BCL‐2 inhibitors, to minimize genotoxic selective pressure [21, 23, 24].

In the case presented in this report, serial tissue biopsies correlated with peripheral blood findings showing a combination of cells with typical CLL morphology and a progressively increasing prolymphocytic population. Also, serial *IgH* gene rearrangement studies by PCR showed the persistence of the same clone despite evolving prolymphocytic morphology. Peripheral blood flow cytometry similarly showed a single distinct monoclonal B‐cell population with a progressive increase in the intensity of the kappa light chain correlating with evolving cytomorphology [3].

Several limitations of this case merit explicit acknowledgment. First, FISH studies, *IGHV* somatic hypermutation analysis, *TP53* mutation sequencing, and next‐generation sequencing for *NOTCH1*, *SF3B1*, and other recurrently mutated genes in CLL were not performed at any time point, reflecting past clinical practice norms. This precludes precise molecular characterization of the drivers of prolymphocytic progression and limits direct comparability with contemporary literature. Second, the axillary lymph node biopsy at Year 12 was not PET/CT‐guided; the retroperitoneal/periaortic mass represented the dominant site of radiological progression and would have been the preferred sampling target for transformation assessment but was deferred on safety grounds given its anatomical relationship with major vascular structures and the patient’s anticoagulation status. The subsequent retroperitoneal biopsy at Year 16, however, corroborated the axillary findings and confirmed the absence of large‐cell transformation. Despite these limitations, the integration of serial morphological, immunophenotypic, and clonality data across multiple time points over 18 years provides a coherent and internally consistent evidence base for the diagnosis of prolymphocytic progression in CLL.

## 4. Conclusions

In summary, this case of CLL with prolymphocytic progression over an 18‐year course highlights the critical role of integrating careful morphologic review, immunophenotypic profiling, and clonality studies to distinguish prolymphocytic evolution from RT or de novo B‐PLL. The patient’s steadily rising prolymphocyte count—accompanied by subtle increases in kappa light chain intensity and stable IgH clonality—marked a shift to a more aggressive disease phase that required successive adaptations in therapy, from chemoimmunotherapy to targeted agents. Recognizing prolymphocytic progression is essential, as it carries distinct prognostic implications and mandates serial peripheral blood smear evaluation, tissue biopsies to exclude large‐cell transformation, and prompt therapeutic adjustment. This report underscores the necessity of ongoing vigilance for atypical morphologic and immunophenotypic changes in CLL, ensuring timely diagnosis and individualized management to optimize patient outcomes.

## Author Contributions

Conceptualization: Rohit Gulati and Magdalena Czader; investigation and data curation: Harini Venkatraman Ravisankar and Liang Chen; writing–original draft preparation: Rohit Gulati; writing–review and editing: Harini Venkatraman Ravisankar, Kavita Umrau, Liang Chen, and Magdalena Czader; supervision: Rohit Gulati and Magdalena Czader.

## Funding

This study received no funding/grant support.

## Disclosure

All authors have read and approved the final version of the manuscript Rohit Gulati, had full access to all of the data in this study and takes complete responsibility for the integrity of the data and the accuracy of the data analysis.

## Ethics Statement

The design of the study was reviewed by Institutional Review Board at Indiana University IRB#23442.

## Consent

Written informed consent has been obtained from the patient’s next of kin to publish this paper.

## Conflicts of Interest

The authors declare no conflicts of interest.

## Data Availability

The data that support the findings of this study are available from the corresponding author upon reasonable request.
